# Clinician Perspectives on Clinical Decision Support for Familial Hypercholesterolemia

**DOI:** 10.3390/jpm13060929

**Published:** 2023-05-31

**Authors:** Hana Bangash, Omar Elsekaily, Seyedmohammad Saadatagah, Joseph Sutton, Paul Johnsen, Justin H. Gundelach, Arailym Kamzabek, Robert Freimuth, Pedro J. Caraballo, Iftikhar J. Kullo

**Affiliations:** 1 Department of Cardiovascular Medicine, Mayo Clinic, Rochester, MN 55905, USA; 2 Department of Information Technology, Mayo Clinic, Rochester, MN 55905, USA; 3 Department of Artificial Intelligence and Informatics, Mayo Clinic, Rochester, MN 55905, USA; 4 Department of General Internal Medicine, Mayo Clinic, Rochester, MN 55905, USA; 5 Gonda Vascular Center, Mayo Clinic, Rochester, MN 55905, USA

**Keywords:** Clinical decision support, CDS, familial hypercholesterolemia, implementation science, stakeholder engagement, electronic health record

## Abstract

Familial Hypercholesterolemia (FH) is underdiagnosed in the United States. Clinical decision support (CDS) could increase FH detection once implemented in clinical workflows. We deployed CDS for FH at an academic medical center and sought clinician insights using an implementation survey. In November 2020, the FH CDS was deployed in the electronic health record at all Mayo Clinic sites in two formats: a best practice advisory (BPA) and an in-basket alert. Over three months, 104 clinicians participated in the survey (response rate 11.1%). Most clinicians (81%) agreed that CDS implementation was a good option for identifying FH patients; 78% recognized the importance of implementing the tool in practice, and 72% agreed it would improve early diagnosis of FH. In comparing the two alert formats, clinicians found the in-basket alert more acceptable (*p* = 0.036) and more feasible (*p* = 0.042) than the BPA. Overall, clinicians favored implementing the FH CDS in clinical practice and provided feedback that led to iterative refinement of the tool. Such a tool can potentially increase FH detection and optimize patient management.

## 1. Introduction

Familial hypercholesterolemia (FH), a relatively common monogenic disorder that affects 1.3 million individuals in the United States, remains significantly underdiagnosed [1,2]. Resulting from a pathogenic variant in *LDLR*, *APOB,* or *PCSK9*, FH is associated with an increased risk of adverse events such as myocardial infarction and stroke [1]. There is a need to increase FH awareness amongst clinicians to enable earlier diagnosis. Clinical decision support (CDS) may increase FH detection when integrated effectively in clinical settings [3]. While clinicians are the primary end-users of CDS [4], they are often not involved in the process of CDS design and development. Lack of clinician involvement can hinder the adoption of CDS in practice, increase the cognitive burden and result in workflow disruptions [5,6]. There is a need to engage with clinicians across all stages of CDS development and to evaluate the CDS once it has been implemented in practice to ensure seamless integration with clinical workflows. Such a CDS tool could have higher adoption rates in clinical practice and facilitate the detection and treatment of individuals with FH. 

Using an experience-based, co-design approach and an implementation science framework, we developed CDS for FH and deployed it at an academic medical center [3,7,8]. The CDS for FH was designed to provide evidence-based recommendations to clinicians at the point of care to identify FH patients, facilitate cascade testing of family members and initiate timely treatment to prevent myocardial infarction. During the process of CDS design and implementation, the aim was to engage with clinicians and seek their feedback at each stage, starting from prototype design and ending at post-deployment, at which point clinician insights were sought on the clinical implementation of the tool. In this report, we describe clinician feedback obtained after the CDS was implemented in the electronic health record (EHR), using an implementation survey, to understand factors that could impact CDS adoption in clinical practice. Survey findings informed iterative refinements to the FH CDS and improved its implementation in the EHR.

## 2. Materials and Methods

This study was conducted from November 2020 to February 2021. In November 2020, the FH CDS was deployed as an active alert in the EHR across all three Mayo Clinic sites and the Mayo Clinic Health System (MCHS) in two formats: a best practice advisory (BPA) and an in-basket alert. Clinicians for whom either the BPA or in-basket alert triggered, were invited to participate in an implementation survey via email. The survey included 25 questions, and for each question, a 5-point Likert scale was used to record the response. Survey data were collected and managed using Research Electronic Data Capture (REDCap) software [9]. The survey was developed from the Consolidated Framework for Implementation Research (CFIR) [10,11] and Implementation Outcome Measures [12,13]. The CFIR is a conceptual framework that can be used to identify multilevel contextual factors that may influence the implementation of an intervention [10,11], while Implementation Outcome Measures can be used to assess whether stakeholders perceive an intervention as acceptable, appropriate, and feasible [12,13]. During an earlier phase of CDS development, this survey was administered to 13 clinicians to gather initial feedback on the prototype, as previously described [3]. 

Descriptive survey data, including the frequency of responses to both alerts, was collated. Using the Mann–Whitney–Wilcoxon Test, we compared responses provided by clinicians for the BPA and in-basket alerts. We also compared responses between different demographic groups, such as male versus female, MDs versus non-MDs, and primary care clinicians versus specialists. A two-sided *p*-value ˂ 0.05 was considered statistically significant. Free-text responses were analyzed using a qualitative, inductive approach. Three research team members (H.B., O.E., and J.H.G.) reviewed each free-text response and categorized it by theme. Survey findings and free-text responses guided iterative refinements to the FH CDS and its clinical implementation. 

## 3. Results

Over three months, survey invitations were emailed to 936 clinicians with a response rate of 11.1% (*n* = 104). This response rate was within the anticipated range and similar to prior reports [2,3,14,15,16]. Of the 104 respondents, 48 completed the survey after having received the BPA alert and 56 after having received the in-basket alert. A total of 44 respondents from both groups also provided free-text responses.

### 3.1. Participant Characteristics

Survey respondents included 56 females (53.8%), 32 non-MDs (30.8%), including nurse practitioners and physician assistants, and 82 clinicians (78.8%) from primary care or general practice. The majority of the respondents were practicing in the MCHS (*n* = 47, 45.2%), which serves communities in Wisconsin, Minnesota and Iowa, followed by Mayo Clinic sites in Rochester (*n* = 34, 32.7%), Arizona (*n* = 12, 11.5%), and Florida (*n* = 11, 10.6%). There were no significant differences in survey distribution between the two groups of respondents (BPA versus in-basket) based on demographic factors, including gender (*p* = 0.355), qualification (MD or non-MD) (*p* = 0.909), department (*p* = 0.868), or location (*p* = 0.123) of practice.

### 3.2. Survey Findings

Survey results revealed that 81% of clinicians who received either the BPA or in-basket alert agreed that CDS implementation was a good option for identifying FH patients at Mayo Clinic, and 78% recognized the importance of implementing the tool in practice (Table 1). When asked whether the tool would improve early diagnosis of FH patients, 72% of clinicians agreed; 67% agreed that the tool would fit well with their existing workflow; 62% agreed that the tool was easy to access and incorporate into their workflow; and 78% agreed that it would help clinicians in identifying and referring or managing FH patients. 

In comparing clinician responses to each of the two alert formats, we found a statistically significant difference (*p* < 0.05) for two of the three implementation outcome measures; clinicians found the in-basket alert format more acceptable (*p* = 0.036) and more feasible (*p* = 0.042) than the BPA. The in-basket format was also deemed more appropriate than the BPA (*p* = 0.054). Survey responses based on CFIR domains revealed a significant difference (*p* = 0.019) in how clinicians responded to questions pertaining to ‘inner setting’, which includes characteristics of the organization such as implementation climate and leadership engagement, with a preference for the in-basket. For the domain ‘intervention characteristics’, which encompasses features of the intervention that could impact its implementation and uptake, and the domain ‘characteristics of individuals’, which includes individual-level factors, such as beliefs and knowledge that could affect implementation, [13] there was a borderline significant difference (*p* = 0.059) with a preference for the in-basket over the BPA. There was no significant difference in clinician responses to questions that pertained to the domains ‘outer setting’ (*p* = 0.419), which covers external factors that could impact implementation, and ‘process’ (*p* = 0.751), which encompasses the phases of implementation such as planning and execution [17].

### 3.3. Thematic Analysis

Thematic analysis of the free-text responses revealed 6 key themes (highlighted in Table 2 with representative quotes).

### 3.4. Refinements to the FH CDS

Survey feedback and thematic analysis led to iterative refinements made to the FH CDS, including: (i) changes to the alert verbiage to make the content clearer and easier to understand, e.g., clarifying that the referral being suggested in the alerts was region specific such that a patient seen in Arizona would be referred to a lipid specialist within their region, with the option to change to a different region if needed; (ii) inclusion of additional laboratory tests in the alert logic to rule out nephrotic syndrome as a secondary cause of hypercholesterolemia; the study team worked with an expert nephrologist to expand the list of laboratory tests that were used to rule out nephrotic syndrome; (iii) enabling clinicians to override the alert without the need to take any specific action, to reduce cognitive burden and allow for a smoother workflow and user experience; and (iv) inclusion of ‘remind me later’ options to dismiss the alert for a limited time. 

Additionally, key institutional stakeholder meetings were held with Cardiology leadership and clinicians providing care to FH patients across the different sites. The meetings aimed to harmonize referral workflows and align FH-related content and order sets within the EHR to ensure that the available information was consistent across the various resources. Content in AskMayoExpert (AME), a Mayo Clinic knowledge resource, was updated, and referral pathways were established across the institution. 

## 4. Discussion

Following the deployment of a CDS tool for FH at Mayo Clinic, we surveyed clinicians to obtain their insights and feedback on its implementation in practice. There is a need to actively involve clinicians, the primary end users of CDS tools, during all stages of development and deployment to ensure the successful adoption of the tool in practice. Clinician feedback can guide improvements to the CDS, its logic, and its EHR integration. In our study, when clinicians were asked specifically about the FH CDS intervention characteristics, they were largely in favor of its implementation to identify FH patients, and they appreciated its value and importance in improving early diagnosis. Feedback from survey respondents led to iterative refinements to the CDS tool.

CDS alerts can improve patient safety, support diagnostic decisions, and facilitate clinical management [18,19]. However, they can also add to the user’s cognitive burden through alert fatigue, especially when a high volume of alerts is received, the alerts are not applicable, or they contain dense information in a time-limited setting [20]. We received similar feedback via the survey question ‘This tool will not increase the time needed with a patient’—only 35% of clinicians agreed with this statement for the BPA format of the CDS. However, the in-basket format garnered a more favorable response, with 61% of clinicians agreeing with the statement. The difference in response to the BPA versus the in-basket alert could be explained by the differing clinical workflow integrations for each alert. The in-basket format provided the clinicians with the patient’s lipid profile results simultaneously with the alert message. It likely resulted in fewer workflow disruptions than the BPA, which triggered passively in the EHR and remained highlighted until it was acted upon by the clinician, leading to an interruption of the usual workflow. The differing alert integration with clinical workflows is likely why the in-basket format was preferred to the BPA format across all survey questions. Through free-text responses, clinicians emphasized the need to ensure the CDS triggered in the right setting at the right time for the right clinicians. 

Our findings indicate that while clinicians largely viewed the FH CDS implementation positively, there is a need to continue to obtain feedback even after deployment of the tool to enable seamless integration with workflows and limit cognitive burden. Clinician feedback at all stages of CDS development should be sought to iteratively refine the alert and its EHR integration. Our survey findings led to changes in CDS content and interface design as well as changes to the linked phenotyping algorithm. Survey findings also resulted in institutional-level discussions to harmonize FH content, resources, and referral pathways. The CDS tool for FH is portable to other hospitals and institutions and, with adoption by clinicians, can increase the diagnosis of individuals with FH and optimize their treatment with guideline-based recommendations. 

A limitation of our study is the modest survey response rate; however, such a response rate lies within the average survey response rate and is similar to that in prior work [2,3,14,15,16]. Thus, it was deemed adequate for this study. Additionally, as the survey targeted practicing clinicians with limited time, only a single email invitation was sent with no further reminders so as to limit cognitive burden. Other studies have also found that internet-based surveys of healthcare providers can yield response rates that vary widely, ranging from 9–94% [15].

## 5. Conclusions

Successful adoption of a CDS tool depends on several factors, including clinician uptake and effective integration of the tool with clinical workflows. While clinicians are the primary users of CDS, they are often not included in the process of CDS design and implementation, which can subsequently limit the adoption of the tool in practice. In this study, we obtained clinician feedback after deploying CDS for FH in clinical practice. Our findings highlight the importance of ensuring that after implementing CDS in practice, it is evaluated by key stakeholders, such as clinicians, to determine whether it harmonizes with their clinical workflows. Such an approach can minimize workflow disruptions, reduce the burden caused by complex EHR systems and increase adoption. With effective integration in practice, the CDS for FH can promote awareness of FH amongst clinicians, increase detection of FH patients, and harmonize treatment with guidelines to improve patient outcomes. 

## Figures and Tables

**Table 1:** BPA and in-basket survey results compared across implementation outcome measures and CFIR domains.

Measures and Constructs Assessed	Question	BPA (*n* = 48)		In-Basket (*n* = 56)		*p*-Value
		Completely Agree/Agree *n* (%)	Score Mean ± SD	Completely Agree/ Agree *n* (%)	Score Mean ± SD	
Acceptability of Intervention Measure (AIM)	This tool meets my approval	29 (60%)	3.7 ± 0.9	44 (79%)	4.0 ± 0.9	0.036
	This tool is appealing to me	30 (63%)	3.6 ± 1.0	46 (82%)	4.0 ± 0.9	
	I like this tool	28 (58%)	3.6 ± 1.0	42 (75%)	4.0 ± 1.0	
	I welcome this tool	32 (67%)	3.7 ± 1.0	45 (80%)	4.0 ± 1.0	
Intervention Appropriateness Measure (IAM)	This tool seems fitting	33 (69%)	3.7 ± 0.9	47 (84%)	4.1 ± 0.9	0.054
	This tool seems suitable	32 (67%)	3.7 ± 0.9	47 (84%)	4.1 ± 0.9	
	This tool seems applicable	34 (71%)	3.8 ± 0.9	50 (89%)	4.1 ± 0.8	
	This tool seems like a good match	31 (65%)	3.7 ± 1.0	45 (80%)	4.0 ± 0.9	
Feasibility of Intervention Measure (FIM)	This tool seems implementable	30 (63%)	3.7 ± 1.0	46 (82%)	4.1 ± 0.9	0.042
	This tool seems possible	32 (67%)	3.8 ± 0.8	47 (84%)	4.1 ± 0.8	
	This tool seems doable	32 (67%)	3.8 ± 0.9	47 (84%)	4.1 ± 0.8	
	This tool seems easy to use	28 (58%)	3.6 ± 1.1	41 (73%)	3.9 ± 1.0	
Intervention Characteristics	I trust the quality and validity of evidence supporting this intervention	31 (65%)	3.8 ± 1.0	40 (71%)	4.1 ± 0.9	0.059
	Implementing this tool is a good option for identifying FH patients at Mayo	36 (75%)	3.8 ± 0.9	48 (86%)	3.9 ± 1.0	
	This tool will improve early diagnosis of patients with FH	32 (67%)	3.5 ± 1.1	43 (77%)	3.9 ± 1.1	
Outer Setting	This tool meets my needs to provide needed resources to my patients	28 (58%)	3.9 ± 0.9	41 (73%)	4.0 ± 0.8	0.419
Inner Setting	This tool is appropriate for Mayo clinicians	34 (71%)	3.9 ± 0.8	45 (80%)	4.0 ± 0.9	0.019
	This tool fits within my existing workflow	30 (63%)	3.5 ± 1.2	40 (71%)	3.9 ± 1.0	
	This tool will not increase the time needed with a patient	17 (35%)	3.0 ± 1.2	34 (61%)	3.6 ± 1.1	
	The implementation of this tool within Mayo is important	35 (73%)	3.8 ± 0.9	45 (80%)	4.2 ± 0.8	
	I recognize the importance of implementing this tool into the practice	37 (77%)	3.9 ± 0.7	44 (79%)	4.0 ± 0.9	
	This tool appears easy to access and incorporate into my workflow	26 (54%)	3.5 ± 1.1	39 (70%)	3.9 ± 1.0	
Characteristics of Individuals	This is a valuable tool for Mayo clinicians	34 (71%)	3.8 ± 0.8	43 (77%)	4.0 ± 0.9	0.059
	This tool will help me identify and refer or manage FH patients	36 (75%)	3.8 ± 1.0	45 (80%)	4.1 ± 0.9	
Process	It is important to me that clinicians at Mayo continue to vet this tool	39 (81%)	4.1 ± 0.8	46 (82%)	4.2 ± 0.9	0.751

**Table 2:** Clinician free-text survey responses identified 6 themes with representative quotes.

Theme	Clinician Feedback	Representative Quotes
Patient preferences on management	Described how their patients responded when the CDS was shared with them during the clinical encounter and the subsequent decisions that were made	*“This particular patient unfortunately declined any additional evaluation or medication, but we did negotiate a follow-up future appointment.”*
Cognitive burden	Recommended that there was a need to recognize alert fatigue and time constraints when implementing the CDS in practice	“*I think it’s important, accurately identifies those at risk, but I am not sure how to start the conversation without adding a significant amount of time (for example, when the visit is for leg pain/15 min appointment and this discussion comes up*.”
Clinician perspectives	Discussed usefulness of the alerts for clinicians Gave feedback on how patients were likely to respond to the clinical recommendations in the CDS	*“Worked well. Immediately caught my attention. Patient was referred to Prev Cards [Preventive Cardiology]”* *“My recently identified patient has little to no insurance and may struggle to participate with recommended cares.”*
Clinical workflow	Described how the CDS alerts impacted clinical workflow	*“The time it came up I had already had a conversation with the patient about this, had a plan in place and yet had to go through the process of figuring out how to address the BPA… It represented a significant hindrance on my workflow…”* *“It was helpful to have the information pop into my inbox with results. It took a bit of extra work to provide the referrals, discuss with patient, and implement.”*
CDS Implementation	Provided feedback on the CDS backend electronic phenotyping algorithm Expressed concerns with the hard stop built into the alerts that required forced action to close them Expressed feedback on navigating the FH smart set embedded within the BPA Emphasized need for institutional champions and clinician education on FH and the CDS alerts	*“I believe the one time I saw or remember seeing this tool "pop up" during a patient encounter, it was with a patient with Nephrotic Syndrome and not necessarily applicable.”* *“Difficult to override in Epic.”* *“Quicker links to ordering from the alert [BPA]”* *“I feel like more education for primary docs would be helpful to make sure we understand what this is and why we are doing that.”*
Usability	Provided suggestions on how to improve the alert content and interface design Recommended that CDS content and the FH smart set align with institutional knowledge resources and order sets	*“Is there a way to condense the information? It just feels a bit overwhelming”* *“The labs recommended by this tool prior to lipid consult in Cardiology do not match the labs recommended prior to an appointment for FH in the Cardiology order set.”*

## Data Availability

The data presented in this study are available on request from the corresponding author.
